# Dental Surgery

**Published:** 1890-09

**Authors:** 


*Dental Surgery*. By HENRY SEWILL, M.R.C.S., L.D.S. Eng. Third Edition. London: BAILLIÈRE, TINDALL & COX. 1890.

This work has been entirely remodelled and very much enlarged, greatly to its advantage. It does not profess to replace the larger text-books of dental surgery; yet it may be read with advantage by every student, who at the commencement of his work can have nothing better for providing him with a theoretical basis for the practical work with which he will be occupied later. As a book of reference for the general practitioner it is excellent. It is all well arranged, the subjects being dealt with in a sequence and way which make reference easy.

The chapter on Caries, the author's special subject, is really a very scientific and thorough treatise, containing the result of much original research. The photo-micrographs are scientific and beautiful additions to the early chapters. The book is well done in every way.

